# Exome sequencing link mutation in *RGPD4* with systemic sclerosis-associated interstitial lung disease and the low level of testosterone—an exploration study

**DOI:** 10.3389/fonc.2022.956552

**Published:** 2022-09-08

**Authors:** Na Wang, Qian Zhang, Wei Sun, Xiaoyu Yang, Hui Huang, Zuojun Xu

**Affiliations:** ^1^ Department of Critical Care Medicine, Peking Union Medical College Hospital, Chinese Academy of Medical Sciences, Beijing, China; ^2^ Department of Respiratory and Critical Care Medicine, Peking Union Medical College Hospital, Chinese Academy of Medical Sciences, Beijing, China

**Keywords:** RGPD4, systemic sclerosis, interstitial lung disease, testosterone, whole exosome sequence

## Abstract

**Background:**

Interstitial lung disease (ILD) is the most common and potentially most devastating manifestation of SSc in pulmonary involvement. However, the mechanism for systemic sclerosis-associated ILD (SSc-ILD) is unclear. This work aims to explore the potential candidates for SSc-ILD upon whole exome sequencing (WES) and attempts to analyze the possible pathogenesis of SSc-ILD from the perspective of the genetic level.

**Materials:**

Variants were confirmed by whole exome sequencing (WES), and SKAT analysis was employed to explore the most differential variants. Targeted variants were performed in biological functions, associated with clinical manifestations, and the probable change of downstream.

**Results:**

By WES and SKAT analysis of SSc with and without ILD, only the variants of *RGPD4* achieved statistical power (*P* < 2.51 × 10^-6^, P-FDR = 0.025, OR = 15.95). A total of 20 rare functional variants (missense, truncating, splicing) were tested for the *RGPD4* gene, and five truncating and damaging missense variants were identified. Carriers showed the older inclusion age (*P* = 0.02) and the higher frequency use of prednisone (*P*=0.02) compared to the non-carriers. Further analysis illustrated that carriers showed lower levels of TES in comparison to non-carriers but did not reach statistical difference (*P* = 0.08). In bivariate correlation analysis, we analyzed the relationship between the mutant status of *RGPD4* and the levels of sex hormones after adjusting for age confounders. Only the level of TES showed a negative correlation with the mutant status (B = -0.509, *P* = 0.037).

**Conclusion:**

The variants of *RGPD4* might contribute to the ILD development of SSc and might also be a causative factor of lower TES among SSc-ILD, which provided insight to a better understanding of pathobiology of SSc-ILD, and androgen hormone supplement might be a therapeutic target in this debilitating disease.

## Background

Systemic sclerosis (SSc) is a refractory connective tissue disease characterized by localized or diffuse skin thickening, progressive organic fibrosis, and ongoing vasculopathy (1). Pulmonary involvement is a common extra-skin manifestation of SSc, and interstitial lung disease (ILD) serves as the leading cause of death (2). However, the exact mechanisms of SSc-associated ILD (SSc-ILD) are unclear. Multiple genetic, epigenetic, and environmental factors contributed to the onset of SSc, and emerging evidence suggests that dysbiosis in the lung, skin, and gastrointestinal tract might trigger the chronic inflammation and autoimmunity in specific organs (3). Moreover, digital vasculopathy, known as Raynaud’s phenomenon, is the most common presenting feature and may precede diagnosis by many years (4).

A genetic component has been well established, with most risks attributed to variants in the immunological pathways including cell-mediated (*via* MHC) and innate (*via* TLRs) immune activation. Moreover, multiple studies suggest that genetic variations have been implicated in specific phenotypes of SSc-ILD besides disease susceptibility. The minor allele of *IRF5* rs4728142:G>A was associated with better survival (*P* = 0.002) and mild ILD (*P* = 0.019) after adjusting age, gender, cutaneous involvement, and duration of disease (5). Some variants were reported to be correlated with specific clinical predictors, like *STAT4* rs757486 with FVC% pred (6), *CTGF* G-945C with ATA positive, and *SOX-5* rs11047102 with ACA positive (7). For these candidates, it is believed that loss-of-function (LoF) variants might be the culprit of genetic disorders.

To explore the genetic etiology of SSc-ILD and uncover a novel mechanism of pulmonary development, we have applied whole exome sequencing to search the potential candidates in 80 patients with or without ILD and 208 healthy controls. We initially provide evidence that *RGPD4* variants might participate in the development of SSc-ILD in comparison to the other two groups upon SKAT analysis (*P* = 8.13 × 10^-7^, P-FDR = 0.015, OR = 17.23), especially LoF with a severe ILD phenotype (radiological pattern: UIP). Moreover, through biological function exploration of *RGPD4*, including the GTEx, GO, PPI, and GSCA Lite databases, it was reported that *RGPD4* with the other four interactive proteins participated in the modulation of hormone receptors. Moreover, our data presented that *RGPD4* variants might correlate with lower levels of testosterone in patients with SSc-ILD.

RANBP2-like and GRIP domain-containing 4 (RGPD4) is a protein-coding gene, and diseases associated with *RGPD4* involve ovarian serous cystadenocarcinoma (OMIM 612707). Currently, few researchers explored the physiological function of this gene. A study in combination of the computational oncoproteomics approach and analysis of genetic expression with patient survival revealed that *RGPD4* was a significantly mutated protein in OV (*P* = 0.0.002), and higher levels of *RGPD4* protein indicated poor survival in LUAD (*P* = 0.048) (8). In the GeneCards database, GenomeRNAi human phenotypes for *RGPD4* included G0/1 arrest, strongly decreased NFAT1 nuclear translocation, decreased viability, and increased gamma-H2AX phosphorylation (https://www.genecards.org). The nuclear factor of activated T cells (NFAT), a key regulator of T-cell development and function, could mediate the IL-2 related immunological pathway; meanwhile, its nuclear translocation was proved to be correlated with HIF-α-induced fibroblast fibrosis (9).

Women of childbearing age owned priority in the incidence of systemic sclerosis (10), and it is well known that sex hormone status regulated a few immunomodulatory functions (11). Luisa et al. (12) reported that an altered androgen could result in relative immunological hyperactivity contributing to enhance tissue damage and disease severity in SSc. Moreover, our team reported that testosterone showed a strong correlation with idiopathic pulmonary fibrosis (13). However, data on levels of sex hormones and their role in ILD are scare.

Thus, the objectives of this study were (i) to explore a pilot study that compares differential genes measured by WES and SKATO analysis in patients with SSc-ILD and patients with SSc without ILD and (ii) to present the biological function of potential candidates of SSc-ILD that compass the clinical relevance of patients with SSc-ILD.

## Materials

### Cohort collection

**Figure 1:**
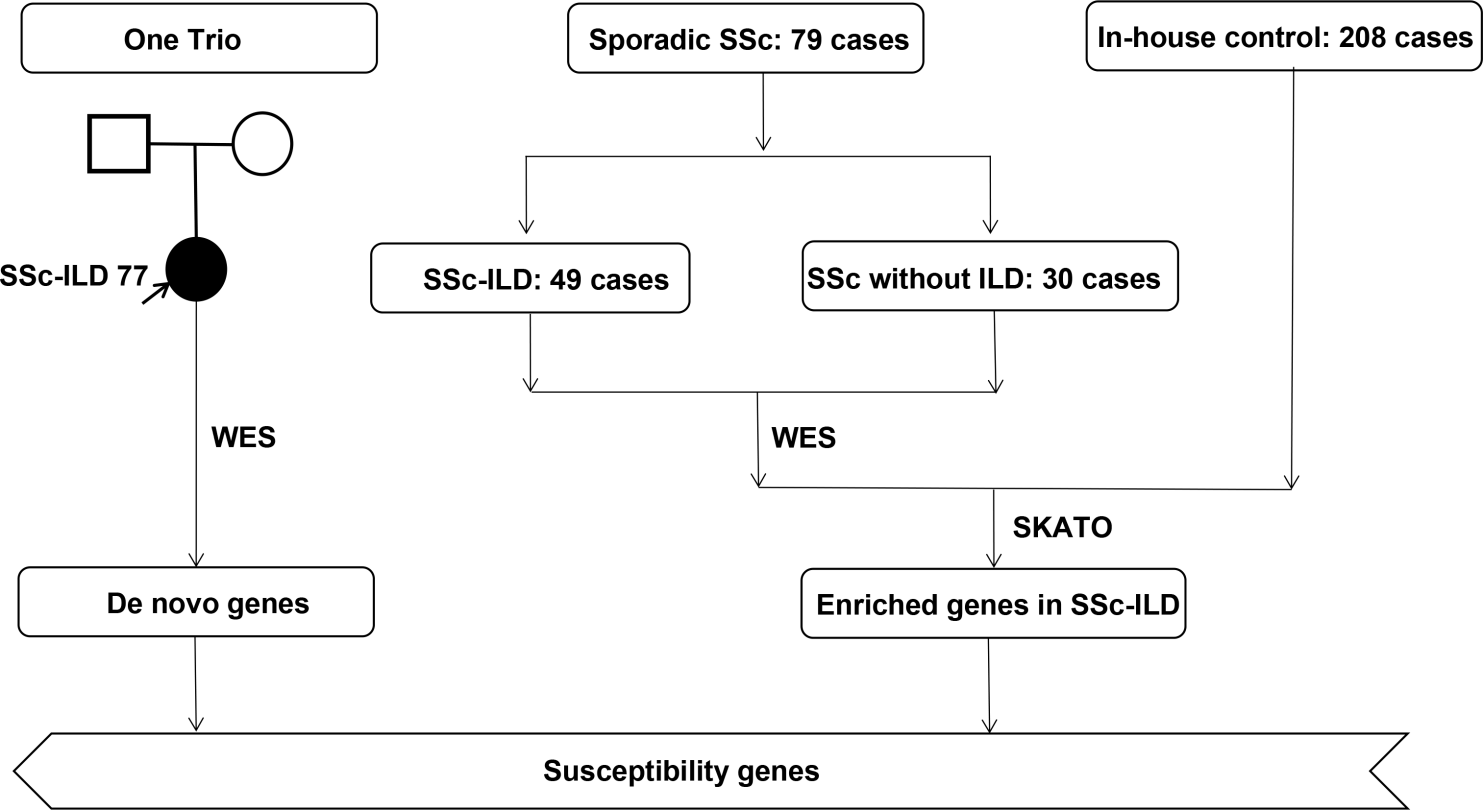
An overview of the prioritization workflow.

This was a retrospective, case–control study that was strictly carried out after getting the written informed consent from all participants and being approved by the ethics committee of Perking Union Medical College Hospital [JS1127]. Consecutive patients with SSc or SSc-ILD hospitalized in Perking Union Medical College Hospital (PUMCH) between January 2015 to January 2021 were retrospectively enrolled, and all the peripheral blood samples were collected at the time of hospitalization. A total of 80 cases, including 50 cases with SSc-ILD and 30 cases with SSc without ILD, and 208 healthy controls were enrolled in this study **Figure 1**. All included cases met the 2013 European League Against Rheumatism (EULAR) and the American College of Rheumatology (ACR), including immunological, fibrotic, and vascular features of the disease, and patients with a total of score greater than 9 are classified as having definite systemic sclerosis (1).

All SSc patients underwent chest CT examination for the evaluation of ILD, which were classified as UIP, possible UIP, NSIP, or possible NSIP according to the international standard (2) (14, 15). UIP is defined as reticular, honeycombing, and traction bronchiectasis and architectural distortion that distributed in the peripheral, subpleural, and basal lobes. NSIP is featured by ground glass opacity (GGO), irregular lines, and consolidation that also distributed in the peripheral, subpleural, and basal lobes. Moreover, Orlandi and colleagues concluded that the features in CT images of SSc-ILD were fibrosis inside focal GGOs in the upper or lower lobes and reticulations in the lower lobes (especially if bilateral and symmetrical). Each diagnosis of ILD was combined with clinical characteristics, pulmonary function tests, and HRCT findings. HRCT findings were defined as ILD by consensus between radiologists and pulmonologists. Those with known environmental exposures, specific drug-induced pneumonia, or other known causes of ILD were excluded.

### Whole exome sequencing

Peripheral blood samples were extracted from patients after obtaining informed consent. Every patient provided 1 ml of peripheral blood, and all the samples were stored at -80°C until further analysis. Exome sequencing was performed on DNA extracted from peripheral blood from 80 cases and 208 healthy controls, totaling 288 subjects. A series of quality control, DNA modification, end repair, adapter ligation, size selection, and PCR amplification were performed, and then high-throughput sequencing was performed on Illumina PE150 according to a previous study (16).

### WES data interpretation

All tested SNPs and Indels needed quality control to ensure reliability, including GATK standard, Hardy–Weinberg equilibrium, and sample missing rate. Additionally, a principal component analysis was conducted to assess the effect of population stratification. Variants with high allele frequency (minor allele frequency >1%) were excluded from the analysis.

Sequence Kernel Association Test-Optional (SKAT-O) were performed assuming potential candidates among different groups. In our SKAT-O analysis, a total of 18,899 genes were included and genes with a P value of less than 2.51 × 10^-5^ were defined as the effective *P* value.

### Sex hormone measurements

Under the condition of gender and age matching, a total of 96 postmenopausal women were enrolled to detect the concentration of sex hormones, including 17 patients with SSc-ILD, 9 patients with SSc without ILD, and 70 healthy people. All subjects provided 1 ml of peripheral blood, and plasma DS, TES, E2, and PRL levels were measured by enzyme-linked immunosorbent assay (ELISA) after centrifugation for plasma. All reagents used are commercially available (Novus Biologicals, Colorado, USA).

### Statistical analysis

All data were analyzed using SPSS 25.0 software (IBM Statistics, Chicago, IL). Categorical variables were presented by percentages, and continuous data were presented as means and SD. Differences in categorical variables were analyzed using Fisher exact test, and differences in the continuous data were analyzed by Student’s t-test and ANOVA test. It was considered statistically significant when the *P* value was under 0.05.

## Results

### Cohort information

We enrolled a total of 30 cases with SSc without ILD, 49 cases with SSc-ILD, and one case with unaffected parent (trio) of Chinese Han ethnicity, consisting of 67 women and 13 men. Patients with SSc of late-onset year, longer duration, and positive anti-Scl-70 were of priority in suffering from interstitial lung disease. A total of 47 SSc patients suffered from microvascular damage, including Raynaud’s phenomenon (RP), pulmonary arterial hypertension (PAH), scleroderma renal crisis, and gastric antral vascular ectasia, and SSc-ILD patients had a higher risk than SSc patients without ILD [*P* = 0.03, OR 2.78, 95% CI (1.09-7.08)]. RP is the most common vascular damage of SSc patients, and ILD patients had a higher risk of PAH than patients without ILD among SSc patients [*P* = 0.02, OR 1.19, 95% CI (1.05-1.34)]. Moreover, higher levels of infectious and inflammatory indicators were found in patients with pulmonary involvements, like ESR (*P =* 0.004) and NLR (*P* < 0.001). Interstitial lung disease, as a serious complication, required a combination of hormone and immunosuppressive therapy in the clinical process compared to those without complications (*P =* 0.040). A total of six types of immunosuppressants were involved, and two antifibrotic agents were used among the 80 patients, and CTX is the most favorable immunosupressant. Detailed demographic information is presented in **Table 1**.

**Table 1:** Demographic and phenotypic characteristics of systemic sclerosis (SSc) vs. systemic sclerosis-associated interstitial lung disease (SSc-ILD).

Characteristics	All (N = 80)	SSc without ILD (N = 30)	SSc-ILD (N = 50)	*P*value
Female sex—no./total no. (%)	67 (83.8)	25 (83.3)	42 (84.0)	1.000
Age at inclusion—year	47.9 ± 15.8	38.7 ± 17.3	53.3 ± 12.1	<0.001^*^
SSc duration—year	4.9 ± 4.2	3.1 ± 2.2	5.8 ± 4.7	0.009^*^
ILD duration—year			4.4 ± 2.3	
Microvascular damage	47 (58.8)	13 (43.3)	34 (68.0)	0.030
RP	36 (45.0)	12 (40.0)	24 (48.0)	0.486
PAH	8 (10.0)	0	8 (16.0)	**0.022**
Scleroderma renal crisis	2 (2.5)	0	2 (4.0)	0.525
Gastric antral vascular ectasia	1 (1.25)	1 (3.3)	0	0.375
Laboratory indicators				
Positive anti-Scl-70	33 (41.2)	6 (20.0)	27 (54.0)	0.004^*^
Positive ACA	4 (5.0)	2 (6.7)	2 (4.0)	0.628
ESR	14.4 ± 15.3	8.4 ± 5.7	18.0 ± 17.9	0.004^*^
hsCRP	3.0 ± 5.7	2.1 ± 6.2	3.7 ± 5.4	0.358
NLR	3.0 ± 1.8	2.2 ± 0.8	3.6 ± 2.0	<0.001^*^
Pattern of high-resolution CT				
NSIP or possible NSIP-no./total no. (%)	43 (53.8)		43 (86.0)	
UIP or possible UIP-no./total no. (%)	7 (8.8)		7 (14.0)	
Pulmonary function				
Forced vital capacity—% of predicted value			79.6 ± 17.2	
DLCO% of predicted value			52.1 ± 13.1	
Total lung capacity—% of predicted value			78.2 ± 14.2	
Treatments				
Traditional Chinese medicine	28 (35.0)	14 (46.7)	14 (28.0)	0.098
Pred	13 (16.3)	6 (20.0)	7 (14.0)	0.533
Pred+immunosuppresants	39 (48.8)	10 (33.3)	29 (58.0)	0.040^*^
CTX	19 (48.7)	5 (50.0)	14 (48.3)	0.925
AZA	3 (7.7)	1 (10.0)	2 (6.9)	0.751
MMF	6 (15.4)	1 (10.0)	5 (17.2)	0.584
FK-506	1 (2.6)	0	1 (3.4)	0.744
MTX	4 (10.3)	2 (20.0)	2 (6.8)	0.267
HCQ	2 (5.1)	1 (10)	1 (3.4)	0.452
CTX+MMF	3 (15.4)	0	3 (10.3)	0.556
CTX+HCQ	1 (2.6)	0	1 (3.4)	0.744
PFD	7 (8.8)	0	7 (14.0)	0.158
Nintedanib	2 (2.5)	0	2 (4.0)	0.584

SSc, systemic sclerosis; ILD, interstitial lung disease; anti-Scl-70, anti-topoisomerase antibody; ACA, anti-centromere antibody; RP, Raynaud’s phenomenon; PAH, pulmonary arterial hypertension; ESR, erythrocyte sedimentation rate; hsCRP, hypersensitive c-reactive protein; NLR, neutrophil/lymphocyte ratio; NSIP, non-specific interstitial pneumonia; UIP, usual interstitial pneumonia; DLco, diffusion Capacity for carbon monoxide; Pred, prednisone. The bold values means statistical significance. The colored text in Table 1 means statistical significance (P<0.05).

### Association of RGPD4 mutation with risk of SSc-ILD

**Figure 2:**
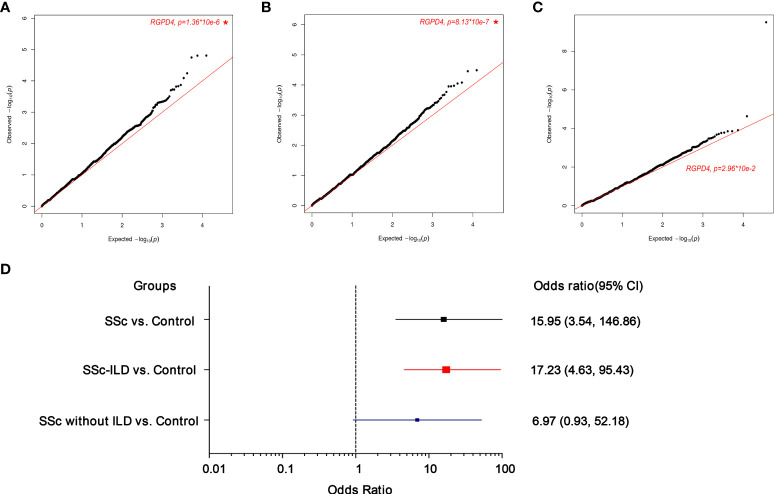
Quantile–quantile(q-q) plot of p values for rare variants test of association between each gene and the presence of the systemic sclerosis (SSc) phenotype upon SKATO analysis (cases=80, controls=208). A total of 18899 genes with at least 4 rare variants were included for correlation with a risk of SSc or ILD. The star indicates the most significant gene. **(A)** SSc vs. Control **(B)** SSc-ILD vs. Control **(C)** SSc without ILD vs. Control **(D)**. The forest plots mainly shows the odds ratio(OR) and 95% confidence intervals (CI). The box indicates the OR value.

In order to explore novel pathogenic genes potentially contributing to the ILD of SSc, whole exome sequencing (WES) was performed. A total of 18,899 genes were screened to execute a SKAT-O analysis, and the top 20 genes identified with *P* < 1 × 10^-3^ were demonstrated in each group in **Table 2**. In the comparison of the SSc group (n = 80) and control group (n = 208), only *RGPD4* on chromosome 2q12.3 met the *P*-value threshold criteria (0.05/18,899 = 2.51 × 10^-6^) (*P =* 1.36 × 10^-6^, P-FDR = 0.025, OR = 15.95). The patients were subsequently divided into patients with SSc-ILD and patients with SSc without ILD. Next, this outcome remained unchanged in the SSc-ILD group (n = 50) vs. control group (n = 208) (*P* = 8.13 × 10^-7^, P-FDR = 0.015, OR = 17.23) rather than the SSc without ILD group (n = 30) vs. control group (n = 208) (*P* = 2.96 × 10^-2^, P-FDR = 0.82, OR = 6.97) (**Figure 2**) , and the *P-*value and OR value showed the most significance in the SSc-ILD group.

**Table 2:** The top 20 genes sorted by SKAT-O analysis by comparison among the three groups.

SSc (n = 80) vs. control (n = 208)			SSc-ILD (n = 50) vs. control (n = 208)			SSc without ILD (n = 30) vs. control (n = 208)		
Gene	β-weighted P^*^	*P*-FDR^#^	Gene	β-weighted P^*^	*P*-FDR^#^	Gene	β-weighted *P* ^*^	*P*-FDR^#^
**RGPD4**	**1.36E-06**	**0.02570314**	**RGPD4**	**8.13E-07**	**0.015385293**	FRG2EP	3.17E-10	5.99E-06
LOC100507053	1.55E-05	0.084454348	KRT86	3.24E-05	0.220461693	LOC441242	2.34E-05	0.220692353
NBPF25P	1.57E-05	0.084454348	RIMBP3	3.50E-05	0.220461693	LGALS13	0.000123211	0.443729062
KRT86	1.79E-05	0.084454348	LGSN	8.32E-05	0.264412724	KMT5C	0.000139233	0.443729062
FBL	5.71E-05	0.21599061	ADSSL1	8.92E-05	0.264412724	POM121C	0.000140012	0.443729062
FSCN3	8.11E-05	0.255535945	ANGPTL2	0.000106123	0.264412724	PSPH	0.000165	0.443729062
FOCAD	0.000135153	0.313961688	CYP2D7	0.000110771	0.264412724	ACD	0.000171069	0.443729062
HAUS7	0.000147608	0.313961688	SCN5A	0.000111832	0.264412724	RGS6	0.000194072	0.443729062
CELA3A	0.000152003	0.313961688	LOC100507053	0.000170196	0.35769435	FCGR1B	0.000211367	0.443729062
ANGPTL2	0.000188731	0.313961688	NBPF25P	0.000209921	0.378451073	WDR89	0.000262985	0.450841956
FATE1	0.000188731	0.313961688	AKT1	0.000220088	0.378451073	CEACAM4	0.000295345	0.450841956
LGI2	0.000199351	0.313961688	FBL	0.00026862	0.423411748	FOXS1	0.000317532	0.450841956
KDELC1	0.000313564	0.380218573	PRDM7	0.00029229	0.425281713	BMX	0.000332387	0.450841956
ITGA9	0.000354584	0.380218573	CLEC10A	0.000343862	0.431280774	MFSD7	0.000334063	0.450841956
PDCD2L	0.000389436	0.380218573	CELA3A	0.000376614	0.431280774	SUSD4	0.000418106	0.51241795
RPH3A	0.000423572	0.380218573	RPH3A	0.000387449	0.431280774	LOC100507053	0.000457918	0.51241795
CEACAM4	0.000434091	0.380218573	SERPINA4	0.000393691	0.431280774	KREMEN2	0.000490583	0.51241795
SUSD4	0.00045295	0.380218573	CCDC63	0.000455853	0.431280774	PRDM14	0.000495478	0.51241795
ADSSL1	0.00045689	0.380218573	VSIG4	0.00046778	0.431280774	CTSF	0.000515293	0.51241795
YY1	0.000468041	0.380218573	UBE2Q2L	0.000502863	0.431280774	RGPD4	0.02962771	0.821429126

^*^P values from the β-weighted sequence kernel association test with optimal kernel weighting. ^#^P values from the false discovery rate (FDR).The bold values means statistical significance.

### The clinical manifestation of patients carrying RGPD4 mutation

**Figure 3:**
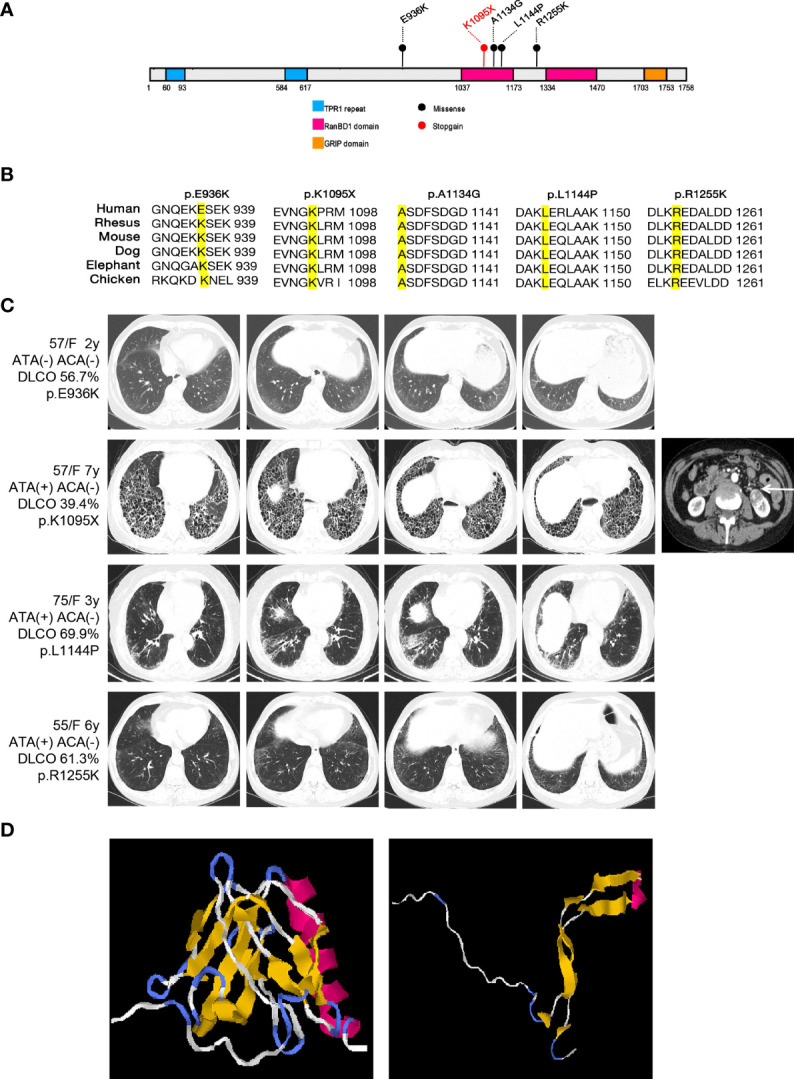
Overview of the damaging variants of RGPD4 and the radiological features of patients carrying the corresponding variants. **(A)** Schematic of the functional domains of RGPD4 protein with the damaging variants indicating by the circles. The blue rectangles indicate the TRP1 repeat domain. The red rectangles indicate the RanBD1 domain. The orange rectangle indicates the GRIP domain. The black indicates the missense and the red indicates the stopgain. **(B)** Alignments of RGPD4 amino acid sequences from Human, Rheusus, Mouse, Dog, Elephant and Chicken. **(C)** The radiological manifestation of the patients carrying the damaging variants of RGPD4. **(D)** Predicted 3D structure of normal and mutant RGPD4 protein. The left shows the normal structure and the right shows the structure caused by stopgain variation (p.K1095X).

A total of 20 rare functional variants (missense, truncating, splicing) were tested for the *RGPD4* gene, and five patients carried five truncating and damaging missense variants, of which three mutations are located in the RanBD1 domain (**Table 3**; **Figure 3A**). These five truncating and damaging missense variants owned well conservative except p.E936K. (**Figures 3A, B**). While the allele frequency of *RGPD4* did not reach significant difference between the SSc-ILD group (8.0%) and the SSc without ILD group (3.3%) (*P* = 0.645), its highest ranking encouraged us to assess the corresponding phenotypes of carriers and additional datasets to determine its correlation with the development of SSc-ILD (**Table 4**).

**Table 3:** Truncating and damaging missense variants in RGPD4.

Variant type	Variant location	cDNA change	Protein change	gnomADAF	CADD	MutationTaster	SSc-ILD(n = 50)	SSc withoutILD (n = 30)	Control(n = 208)
Missense	2:g.108487266	c.G2806A	p.E936K	1.64 × 10^-5^	NA	1	1 (32)		0
Stopgain	2:g.108487743	c.A3283T	p.K1095X	1.23 × 10^-5^	37	1	1 (61)		0
Missense	2:g.108487861	c.C3401G	p.A1134G	7.07 × 10^-6^	23.5	0.991		1(15)	0
Missense	2:g.108487891	c.T3431C	p.L1144P	0	21.2	1	1 (78)		0
Missense	2:g.108488224	c.G3764A	p.R1255K	2.0 × 10^-4^	23.4	0.973	1 (63)		0

SSc-ILD, systemic sclerosis associated interstitial lung disease; AF, allele frequency; NA, not applicable; CADD, Combined Annotation-Dependent Depletion Score.

**Table 4:** Demographic and phenotypic characteristics of patients with SSc-associated interstitial lung disease (SSc-ILD) according to RGPD4-mutated status.

Characteristics	MT RGPD4 (n = 6)	WT RGPD4 (n = 44)	*P*	OR	95% CI
Female sex—no./total no. (%)	6/50 (12.0)	44/50 (88.0)	0.57		
Age at inclusion—year	59.2 ± 8.3	46.8 ± 12.5	0.02^*^		
SSc duration—year	5.9 ± 3.4	5.9 ± 4.9	0.85		
**Laboratory indicators**					
Positive anti-Scl-70	4 (66.7)	19 (43.2)	0.35	2.63	(0.44,15.91)
Positive ACA	0 (0.0)	1 (2.3)	1.00	0.98	(0.93,1.02)
ESR	18.5 ± 19.1	17.9 ± 18.1	0.95		
hsCRP	2.1 ± 2.5	3.9 ± 5.7	0.59		
NLR	3.9 ± 2.9	3.6 ± 1.9	0.77		
**Pattern of high-resolution CT**					
NSIP or possible NSIP-no./total no. (%)	5 (83.3)	38 (86.4)	0.99		
UIP or possible UIP-no./total no. (%)	1 (16.6)	6 (13.6)	0.99		
**Pulmonary function**					
Forced vital capacity—% of predicted value	88.7 ± 16.7	76.8 ± 17.5	0.23		
DL_CO_% of predicted value	53.9 ± 12.0	53.9 ± 17.1	0.99		
Total lung capacity—% of predicted value	82.8 ± 12.0	77.3 ± 15.4	0.50		
**Treatments**					
Traditional Chinese medicine	1 (16.7)	13 (29.5)	0.66	0.48	(0.05,4.49)
Pred	3 (50.0)	3 (16.8)	0.02^*^	**13.67**	(1.88,99.35)
Pred+immunosuppressants	2 (33.3)	28 (63.7)	0.20	0.29	(0.05,1.73)

SSc, systemic sclerosis; ILD, interstitial lung disease; anti-Scl-70, anti-topoisomerase antibody; ACA, anti-centromere antibody; ESR, erythrocyte sedimentation rate; hsCRP, hypersensitive c-reactive protein; NLR, neutrophil/lymphocyte ratio; NSIP, non-specific interstitial pneumonia; UIP, usual interstitial pneumonia; DLco, diffusion capacity for carbon monoxide; Pred, prednisone.

We observed that these *RGPD4* variants were rare or completely absent from the gnomAD database. Carriers showed female predisposition (100%) and the NSIP radiological pattern (75%) (**Figure 3C**). Notably, a patient carrying the *RGPD4* p.K1095X variant suffered from a severe phenotype, including dyspnea, Raynaud’s phenomenon, UIP radiological pattern, and severe dysfunction of DLCO. Unluckily, the patient suffered from left kidney clear cell carcinoma. In light of the severe phenotype and the stop–gain variation, we subsequently predicted the three-dimensional structure of normal and corresponding mutant RGPD4 proteins. As expected, the findings showed that the spatial structure caused by stop–gain variation was lost in comparison to the normal structure of the *RGPD4* protein (**Figure 3D**).

### The biological function of RGPD4

RGPD4 is a protein-coding gene and is proved to be a candidate gene for ovarian serous cystadenocarcinoma in the OMIM database (612707). It showed no distribution difference in *RGPD4* expression among men and women in the GTEx database, except for testis (www.GTEx.org). The protein–protein interactions (PPI) of *RGPD4* mainly involved four proteins, namely, *SUMO1*, *UBE2I*, *RAN*, and *XPO1* (www.string-db.org). In the GSCALite database, we found that these five interacting proteins showed a strong correlation with malignant diseases, like tumors in the respiratory system (LUSC, LUAD, etc.) and tumors in the urinary system (KICH, KIRC, and KIRP). Furthermore, the mechanism involving such diseases contains the negative regulation of the androgen receptor.

**Figure 4:**
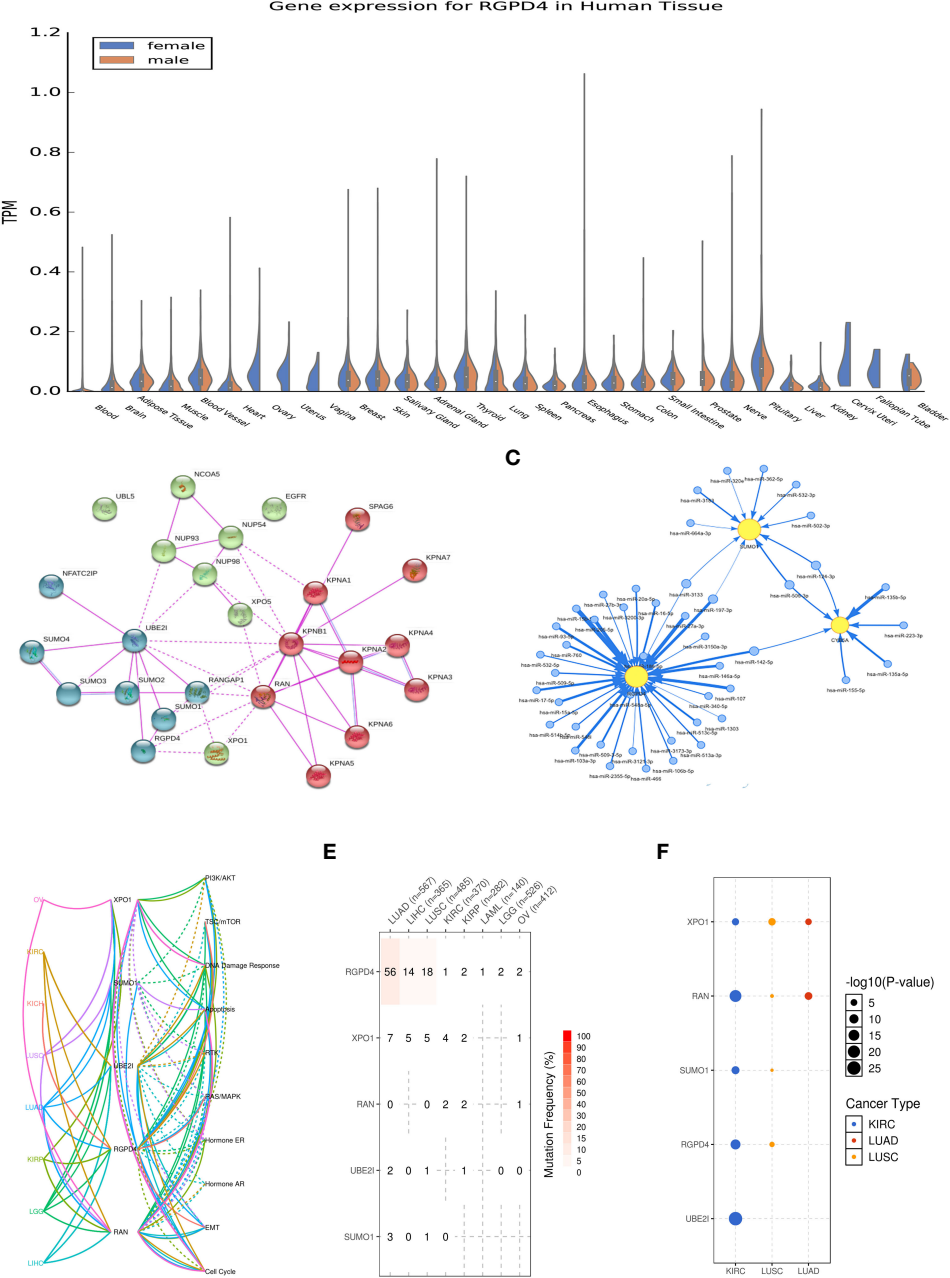
Summary of the biological function of RGPD4. **(A)** Gene expression(in TPM) of RGPD4 in different tissues amongst male and female (www.GTEx.org). **(B)** The protein-protein interactions (PPI) of RGPD4 (www.string-db.org). Line thickness indicates the strength of data support and the medium confidence was 0.4. All the interactions sources derive from experiments. **(C)** The interacted proteins commonly participate in the related pathways of different diseases. KICH, kidney chromophobe; KIRC, kidney renal clear cell carcinoma; KIRP, kidney renal papillary cell carcinoma; LGG, brain lower grade glioma; LIHC, liver hepatocellular carcinoma; LUAD, lung adenocarcinoma; LUSC, lung squamous cell carcinoma; OV, ovary serous cystadenocarcinoma. **(D)** The miRNA network among RGPD4 and the androgen metabolism genes. The blue circle indicates miRNA and the yellow circle indicates gene. **(E)** The reported SNV percentage profile of RGPD4, XPO1, RAN, UBE2I and SUMO1 in different tumors . SNV, single nucleotide variation. **(F)** The reported cancer types of RGPD4, XPO1, RAN, UBE2I and SUMO1. The size of the circle represents statistical difference and the color indicates different cancer.

Further analysis implied that *RGPD4* also showed a correlation with the androgen biosynthesis and metabolism genes, including *CYB5A*, *CYP3A5*, *COMT*, and *STS*. Moreover, the correlation among these genes or proteins mainly involved the microRNAs (**Figure 4**). The abovementioned indirectly provided evidence that *RGPD4* showed a correlation with androgen.

### Potential association between RGPD4 and sex hormone

Systemic sclerosis owns sex priority, as the incidence of the women is 3–14 times higher than the incidence of men (17), and androgen plays a vital role in the development of SSc (18). Additionally, proteomic analysis identified that *RGPD4* determines the occurrence of ovarian serous cystadenocarcinoma, a type of cancer caused by high levels of androgen. Serum proteomic analysis also provided evidence that *RGPD4* expression showed male specificity in Asperger’s syndrome (8). In summary, *RGPD4* showed the sex-specific difference. Thus, we speculate whether *RGPD4* showed a potential correlation with androgen concentration in patients with SSc.

#### The concentration of sex hormone in patients with SSc

**Figure 5:**
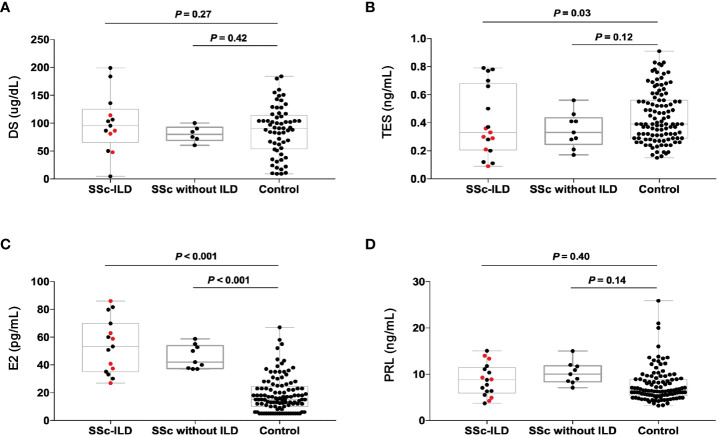
The concentration of sex hormones in the patients with SSc-ILD (n=17), SSc without ILD (n=9) and the healthy people (n=70). The red dots indicate the patients with RGPD4 mutation. **(A)** The DS concentration **(B)** The TES concentration **(C)** The E2 concentration **(D)** The PRL concentration. All the p values are calculated by general linear model after age adjustment. DS dehydroepiandrosterone sulfate, TES testosterone, E2 estrogen, PRL prolactin.

We found that in comparison to healthy control, the TES concentration showed lower levels in patients with SSc-ILD (*P* = 0.03) and the E2 concentration showed higher levels (*P* < 0.001). However, the other two sex hormones, including DS and PRL, showed no significance between cases and control. Furthermore, only the E2 concentration showed higher levels in patients with SSc without ILD than that in healthy control (*P* < 0.001) (**Figure 5**).

We further compare the concentrations of sex hormones in SSc-ILD patients carrying *RGPD4* variants and those who are not. Surprisingly, the levels of TES concentration in carriers appeared to be lower than in non-carriers, but they did not reach statistical difference (0.26 ± 0.09 vs. 0.41 ± 0.25, *P* = 0.08), which might result from the low sample sizes.

#### Associations of variants in androgen biosynthesis and metabolism genes with androgen concentrations

To further explore the mechanism for the lower TES concentration in patients with SSc-ILD, we investigate the variants in androgen biosynthesis and metabolism genes with the levels of TES. A total of 11 SNPs in the coding sequences of androgen biosynthesis and metabolism genes were calculated. The levels of TES among different genotypes did not reach statistical difference after adjustment for age (all *P* > 0.05) (**Table 5**).

**Table 5:** The associations of SNPs in the coding exon of androgen-metabolizing genes with testosterone concentration in patients with SSc-ILD.

Gene	SNP	Genotype^#^	N	Mean	*P*
HSD3B1	rs34638609	AA	13	0.39 ± 0.24	0.332
		AG+GG	4	0.22 ± 0.16	
HSD17B2	rs8191246	AA	15	0.36 ± 0.25	0.682
		AG+GG	2	0.44 ± 0.09	
HSD17B3	rs2066479	CC	8	0.37 ± 0.27	0.962
		CG+GG	9	0.36 ± 0.21	
HSD17B4	rs28943592	CC	13	0.35 ± 0.24	0.486
		CC+CT	4	0.44 ± 0.23	
HSD17B7	rs2684875	AA	12	0.43 ± 0.25	0.072
		AG+GG	5	0.28 ± 0.07	
	rs700519	GG	12	0.36 ± 0.23	0.882
		AG+AA	5	0.38 ± 0.27	
CYP1A1	rs1048943	TT	8	0.40 ± 0.25	0.614
		CT+CC	9	0.34 ± 0.23	
	rs4646422	CC	11	0.37 ± 0.24	0.952
		CT+TT	6	0.36 ± 0.25	
CYP19A1	rs700519	GG	12	0.36 ± 0.23	0.882
		AG+AA	5	0.38 ± 0.27	
COMT	rs6267	GG	14	0.36 ± 0.24	0.764
		GT+TT	3	0.41 ± 0.23	
	rs4680	GG	8	0.31 ± 0.22	0.361
		AG+AA	9	0.42 ± 0.25	

^#^Genotype, ref/alt; HSD, hydroxysteroid dehydrogenase; CYP, cytochrome; COMT, catechin-o-methyltransferase.

#### Potential associations between RGPD4 and androgen concentrations

In bivariate correlation analysis, we analyzed the relationship between the mutant status of *RGPD4* and the levels of sex hormones after adjusting for age confounders. Only the level of TES showed a negative correlation with the mutant status (B = -0.509, *P* = 0.037). Next, after adjusting the mutant status of *RGPD4* and age confounders, the level of TES showed no correlation with the levels of the other three hormones in partial correlation analysis (all *P* > 0.05) (**Table 6**).

**Table 6:** Correlation coefficients between sex hormone and the mutated status of RGPD4 gene as well as correlation coefficients between TES and other hormone after age adjustment.

Variables	RGPD4-mutated status		TES (ng/mL)	
	Coefficients	*P^a^*	Coefficients	*P^b^*
DS (µg/dL)	-0.183	0.439	0.378	0.252
TES (ng/mL)	-0.509	0.037	–	–
E2 (pg/mL)	0.035	0.891	-0.336	0.240
PRL (ng/mL)	0.096	0.687	-0.145	0.593

DS, dehydroepiandrosterone sulfate; T, testosterone; E2, estrogen; PRL, prolactin. Pa: P value was calculated from point-biserial correlation. Pb: P value was calculated from partial correlation analysis causative of lower TES in patients with SSc-ILD, which indicate that the androgen supplement might be potential treatment for patients with SSc-ILD.

## Discussion

Immune-mediated pathways are key in driving the fibrotic process, but how this translates into genetic predisposition is unclear so far. International large cohorts of SSc with and without ILD demonstrated that few specific genetic variants are associated with the development and rate of progression of SSc-ILD. The single-nucleotide polymorphisms (SNPs) of IRF5, IRAK1, CTGF, and CD247 were proved to be the potential candidate genes of SSc-ILD (19). Moreover, the minor allele of IRF5 rs4728142 G>A was proved as a better survival indicator of SSc-ILD (5). Our study introduces the new gene *RGPD4* as a candidate contributing to SSc-ILD upon evaluating SSc patients with and without an ILD phenotype with WES, and five of 80 patients were identified with deleterious or LoF variants of *RGPD4*. Patients carrying those variants showed predominance of lower levels of testosterone than those without variants. Our study provides the potential candidates contributing to SSc-ILD with new insight, which might aid the discovery of new pathogenesis or clinical management for disease.

Interstitial lung disease is a common pulmonary manifestation in patients with SSc and has been the leading cause of death in recent years (20). The 10-year survival rate of SSc was 74.9%, while the 10-year survival rate of SSC-ILD was only 29%–69% (21). It remains an urgent issue to improve clinical outcomes among patients with SSc-ILD. Although new anti-fibrosis drugs, autologous stem cell transplantation, and lung transplantation have been used for treatment, the overall prognosis is not optimistic (22, 23). It still owns great challenge to explore effective and simple treatments. Our team aimed to explore the potential candidates contributing to the pathogenesis of SSc-ILD. The published studies had mostly been executed in subjects of European descent who possess a lower prevalence of ILD than those of African descent (19), and the reported genetic predictors of SSc-ILD mostly derive from common variants; however, the rare variants (minor allele frequency <0.01 or 0.05) are more likely to modulate protein function and result in clinically relevant consequences (24). Our team recently found that the mutant of the gene RANBP2-like and GRIP domain-containing 4 (RGPD4, 2q12.3), one of the members of the RGP family, might be correlated with the pulmonary involvement in SSc patients. Moreover, the variant showed lower levels of testosterone among SSc patients with ILD. In the database of Mouse Genome Information (MGI) upon the mouse model, the phenotypes for the *RGPD4* gene involved the reproductive system phenotype and respiratory disease (http://www.informatics.jax.org). However, these data were proved upon the model of mice rather than human beings and a detailed mechanism is needed.

Another significant question that should be addressed is whether *RGPD4* mutation was related to the level of testosterone or the disease course. Data in the literature demonstrated that androgen steroids play an immunosuppressive role upon the T-cell and B-cell subsets in multiple autoimmune diseases (25). Early studies have found a decreased concentration of DHEAS in SSc patients (16). and Mirone et al. (12)also confirmed that the levels of testosterone and DHEAS correlated with the disease duration and disease severity score among SSc patients. In other autoimmune diseases, including rheumatoid arthritis (RA) and systemic lupus erythematosus (SLE), the level of androgen reflected the severity of systemic damage (26). Our research included the previous research; furthermore, our results implicated that the lower status of testosterone might be modulated by the genetic mutant status in SSc patients with ILD. Our team previously demonstrated that androgen-related pathway genes were associated with the development of IPF by modulating the leukocyte telomere length (13). Whether the lung involvement of SSc patients is related to hormone regulation of leucocyte telomere length needs to be further explored. Interestingly, it had been reported that testosterone supplementation in male SLE patients with genetic hypogonadism alleviated and testosterone protects against the development of joint and lung involvement in male SKG mice (27). The above data, to some extent, provide the hope that androgen replacement therapy may be the new treatment for patients with SSc-ILD.

The major limitation of our study is the small sample size. It was unfortunate that there was insufficient power to test the association between the polymorphism and each of the ILD subsets individually. Next, the animal model of SSc will be built upon the mouse, and the conditional knockout of *RGPD4* in the mouse will be explored to confirm the pathogenicity to interstitial lung disease and detect the level of testosterone. Upon that, the extra testosterone supplement will be administered to evaluate the severity of SSc-ILD. In addition, this research was single-center, and more validation should be performed in the other centers. Lastly, this research mainly attempts to explore the perspective of genomics; the perspective of transcriptome and proteomics further will be explored in the future. Nevertheless, despite these limitations, our study provides important information regarding the potentially distinct pathogenic mechanism involved in patients with SSc-ILD.

## Conclusion

Interstitial lung disease is a common and devastating manifestation of SSc patients and is the leading cause of death in SSc. In order to initiate early recognition and treatment regimens to preclude progressive fibrosis, genetic susceptibility might be involved in the development and rate of progression of SSc-ILD. By searching the potential genetic candidates upon WES and gene burden analysis of SSc with and without ILD, only the gene *RGPD4* variants satisfied the statistical power. Correlation analysis of clinical outcomes and biological function demonstrated that carriers were more prone to suffer from dyspnea, NSIP pattern, and lower TES, which provided insight to a better understanding of the pathobiology of SSc-ILD and androgen hormone supplement might be a therapeutic target in this debilitating disease.

## Data availability statement

The datasets presented in this study can be found in online repositories. The names of the repository/repositories and accession number(s) can be found in the article/supplementary material.

## Ethics statement

The studies involving human participants were reviewed and approved by The Institutional Review Board (IRB) of Peking Union Medical College Hospital (PUMCH). The patients/participants provided their written informed consent to participate in this study.

## Author contributions

ZX contributed to the concept and design of this article. NW mainly participated in the manuscript writing. QZ, Xiaoyan Jing, Jian Guo, and NW participated in the sample collection, sample processing, clinical information collection, and data analysis. ZX and HH analyzed and interpreted the data. All authors read and approved the final manuscript.

## Funding

This work was funded by the National Key Research and Development Plan of Precision Medicine Medical Research (Grant number: 2016YFC0905700). The funding body has no role in the design of the study and collection, in the analysis and interpretation of data, and in the writing of the manuscript. We thank all your support.

## Acknowledgments 

We thank all the patients and their families for participation.

## Conflict of interest

The authors declare that the research was conducted in the absence of any commercial or financial relationships that could be construed as a potential conflict of interest.

## Publisher’s note

All claims expressed in this article are solely those of the authors and do not necessarily represent those of their affiliated organizations, or those of the publisher, the editors and the reviewers. Any product that may be evaluated in this article, or claim that may be made by its manufacturer, is not guaranteed or endorsed by the publisher.
